# Efficient heterogeneous photo-Fenton degradation of azo dyes using the nanocomposite MgFe_2_O_4_/MoS_2_

**DOI:** 10.55730/1300-0527.3796

**Published:** 2026-05-24

**Authors:** Zahra ZEINALI, Leila ERSHADI AFSHAR, Naz CHAIBAKHSH, Farnaz ISAPOUR

**Affiliations:** Department of Applied Chemistry, Faculty of Chemistry, University of Guilan, Rasht, Iran

**Keywords:** Acid Blue 113, azo dye degradation, MgFe_2_O_4_, MoS_2_ nanocomposite, optimization, photo-Fenton

## Abstract

In the present work, a facile hydrothermal route was employed to synthesize the nanocomposite MgFe_2_O_4_/MoS_2_, which acted as a heterogeneous photo-Fenton catalyst for the degradation of a synthetic diazo dye, Acid Blue 113 (AB113). The nanocatalyst produced underwent analysis using multiple characterization techniques. The parameters influencing the oxidative reaction were statistically modeled and optimized using response surface methodology. Optimal degradation efficiency for AB113 (94.92%) was attained at pH 8.79 using 10 mg of MgFe_2_O_4_/MoS_2_ nanocatalyst, 1.6 mL of H_2_O_2_, and a reaction duration of 27 min. The synergistic effect resulting from the formation of electron-hole pairs renders the combined photocatalysis and Fenton process a more efficient treatment approach. The kinetic study data indicate that the photo-Fenton process follows the pseudo-first-order model. The nanocatalyst fabricated demonstrated exceptional stability, maintaining a high degradation performance after seven consecutive photo-Fenton cycles. The results obtained indicate that the MgFe_2_O_4_.MoS_2_/H_2_O_2_/Vis system is an effective method for treating wastewaters containing organic contaminants.

## 1. Introduction

Environmental problems like pollution and water resource depletion have emerged as critical global challenges in recent years [1]. Among these, the contamination of surface and groundwaters by synthetic dyes is a major environmental problem and a potential threat to human health and aquatic ecosystems [2]. Synthetic organic dyes exhibit high structural stability and can remain in water for a long time. These dyes are resistant to light, chemical, and biological treatment because of their complex molecular structure and low biodegradability [3].

Considering the various wastewater treatment approaches developed to date, advanced oxidation processes (AOPs) are recognized as an extremely effective method for dye removal from the environment. AOPs include various technologies such as electrochemical oxidation, photocatalysis, radiolysis, ozonation, sonolysis, and Fenton-related processes [4,5]. The fundamental mechanism of AOPs relies on the formation of reactive oxygen species (ROS), like hydroxyl radicals (^•^OH) and superoxide anion radicals (^•^O_2_^−^), that have strong oxidative potential capable of mineralizing organic contaminants into water and carbon dioxide [6,7].

Among the several advanced oxidation processes, Fenton-type reactions based on the production of hydroxyl radicals have been demonstrated to be effective in degrading organic contaminants. Amongst numerous combinations as heterogeneous Fenton catalysts, spinel ferrites, MFe_2_O_4_ (M = Co, Fe, Ni, manganese, magnesium, copper, zinc, etc.), have garnered much attention as a result of their high catalytic activity, magnetic property, selectivity, and high thermal and chemical stability. In spite of the notable advantages of the Fenton reaction over other AOPs, it is associated with some shortcomings such as low rate and excessive sludge production when used alone [8,9]. Combining ultraviolet or visible light with the conventional Fenton process can enhance the catalyst capacity and degradation efficiency of organic contaminants and decrease iron sludge production [10]. Ultraviolet (UV) or visible light is employed to activate the semiconductors’ surface to generate ROS [11]. Between different semiconductors utilized as materials for photocatalysis, molybdenum disulfide (MoS_2_) shows outstanding features such as a large specific surface area and an appropriate band gap for absorption of visible light [12]. The two-dimensional structure of MoS_2_ renders it a promising photocatalyst, since other nanoparticles can be intercalated between its layers [13]. Furthermore, employing magnetic particles in photocatalytic composite systems can increase the formation of photo-induced charge carriers and their photocatalytic response to visible light [14]. In addition, the combination of metal-substituted ferrites with MoS_2_ creates a dual catalytic effect photocatalyst that is extremely effective. Under light irradiation when H_2_O_2_ is present, the substituted ferrites are able to function as heterogeneous photo-Fenton catalysts [15]. Recent studies on nanocomposites, such as OCN/MnFe_2_O_4_ [16], NiFe_2_O_4_/SiO_2_ [17], MoS_2_/FeVO_4_ [18], MoS_2_/MnFe_2_O_4_ [19], and MoS_2_/BiFeO_3_ [20], have documented the application of ferrites and MoS_2_-based materials in various advanced oxidation processes to remove organic pollutants from wastewaters.

In the present study, the nanocomposite MgFe_2_O_4_/MoS_2_ was synthesized and explored as a photo-Fenton catalyst for the efficient elimination of Acid Blue 113 (AB113), a synthetic diazo dye. Although previous studies have demonstrated the visible-light-responsive nature of MgFe_2_O_4_/MoS_2_, there is no report on its application in a heterogeneous photo-Fenton system for the degradation of AB113 [21].

In the current research, the nanocomposite was prepared via a practical hydrothermal method and analyzed by different analytical techniques. To optimize the operating conditions and reduce experimental cost and effort, response surface methodology (RSM) was employed as a robust statistical and optimization tool for analyzing the interactions among multiple process variables. Finally, the efficiency of removing AB113 was investigated using appropriate analytical methods under the optimized conditions obtained.

## 2. Materials and methods

### 2.1. Chemicals

The chemicals used in the research were purchased from Merck and Sigma-Aldrich and applied without subsequent modification.

### 2.2. Characterization of the catalyst

Fourier transform infrared spectroscopy (FTIR) (Thermo Nicolet Avatar 360 spectrometer) was used for the structural identification of MgFe_2_O_4_, MoS_2_, and MgFe_2_O_4_/MoS_2_ in the range of 400–4000 cm^−1^. The crystal structure and phase of the prepared samples were analyzed by powder X-ray diffraction (XRD) (Philips PW 1730) between 2θ = 10° and 80° using Cu Kα radiation. The catalyst’s morphology was examined using transmission electron microscopy (TEM) (Philips CM120) and field emission scanning electron microscopy (FE-SEM, TESCAN MIRA3), while energy dispersive X-ray spectroscopy (EDS) was applied to determine the existence of elements. The photocatalytic characteristic of the nanocomposite was analyzed using diffuse reflectance spectroscopy (DRS) (model S-4100 SCINCO). The catalyst’s surface area and pore structure were also studied using Brunauer–Emmett–Teller (BET) analysis, and vibrating sample magnetometer (VSM) analysis was utilized to demonstrate the magnetic properties of the fabricated composite.

### 2.3. Synthesis of MgFe_2_O_4_ nanoparticles

In the present work, magnesium ferrite nanoparticles were synthesized via a hydrothermal method. For this purpose, 1.35 g of iron(III) chloride hexahydrate (FeCl_3_.6H_2_O), 0.51 g of magnesium chloride hexahydrate (MgCl_2_.6H_2_O), and 0.02 g of polyvinyl pyrrolidone (C_6_H_9_NO)_n_ were solubilized in 50 mL of ethylene glycol (C_2_H_6_O_2_) while being continuously stirred to produce a uniform mixture. The solution was then vigorously stirred while 3.20 g of sodium hydroxide (NaOH) was gradually introduced. The mixture was stirred continuously for 60 min and after that placed in a Teflon-lined steel autoclave. The suspension was cooled to room temperature after the autoclave was heated to 180 °C for 12 h in an electric oven. The black product was collected by centrifugation and then rinsed three times using ethanol and deionized water. Ultimately, the magnetic powder produced was dried at 80 °C for 6 h and hence the MgFe_2_O_4_ nanoparticles were prepared [22].

### 2.4. Synthesis of MoS_2_

MoS_2_ nanoparticles were created using a hydrothermal method. For this purpose, 20 mL of distilled water was used to dissolve 0.7 g of ammonium heptamolybdate and 1.52 g of thiourea, which were then stirred for 30 min. Thereafter, the suspension was moved to a 100-mL autoclave and heated at 180 °C for 12 h. The precipitate was then dehydrated at 80 °C after being cleaned with ethanol and deionized water, and then ground in a mortar [23].

### 2.5. Synthesis of MgFe_2_O_4_/MoS_2_

To prepare MgFe_2_O_4_/MoS_2_ by a hydrothermal method, 0.7 g of ammonium heptamolybdate tetrahydrate, 1.52 g of thiourea, and 1 g of freshly synthesized magnesium ferrite were dissolved in 30 mL of distilled water. The mixture was stirred for 30 min and after that placed in a Teflon-lined steel autoclave. The autoclave was heated to 180 °C in an electric furnace for 12 h and then the reaction mixture was cooled down to room temperature. The resulting precipitates were rinsed with deionized water and ethanol and dried at 80 °C, and accordingly MgFe_2_O_4_/MoS_2_ was prepared [23].

### 2.6. Photo-Fenton catalytic reactions

The photo-Fenton catalytic performance of the fabricated nanocomposite was assessed via AB113 degradation in the presence of visible light illumination. AB113 was solubilized in distilled water to create a dye solution having a concentration of 25 mg L^−1^. The solution’s pH was adjusted by adding dilute HCl or NaOH and monitored with a pH meter (AZ Instrument Corp., Taichung City, Taiwan, model 86502). Depending on the experimental design, different dosages of MgFe_2_O_4_/MoS_2_ were added to 50 mL of the solution in a 100-mL flask. Prior to irradiation, the sample was stirred in the dark for 15 min to reach absorption equilibrium. Various amounts of H_2_O_2_ were included in order to start the reaction. After that, the sample was put inside a photochemical reaction system with ventilation. Visible light irradiation was provided by a 50-W white LED lamp. The mixture was kept under magnetic agitation during irradiation for a specific duration based on the design of the experiments. Following the reaction’s completion, the suspension was centrifuged at 3000 rpm for 5 min and Whatman filter paper (with pores of 0.22 μm) was used to filter the supernatant to separate MgFe_2_O_4_/MoS_2_. Using a spectrophotometer (JENWAY-7315) set to the maximum absorption wavelength of 595 nm, the dye concentration was measured. The following equation was used to determine AB113 removal efficiency:


(1) $$AB113\;removal\;(\%)=[A_{0}-A]/A_{0}\times 100$$

Here A denotes the average absorbance value at 595 nm following the photo-Fenton oxidation and A_0_ is the value prior to the degradation procedure.

### 2.7. Experimental design and statistical evaluation

A Box–Behnken design was implemented for the experimental design and statistical evaluation of the heterogeneous photo-Fenton oxidation process. Statistical evaluations were performed using Design-Expert 13.0.5 (Stat-Ease Inc., Minneapolis, MN, USA). Numerous factors, such as pH (4.0–9.0), nanocatalyst dosage (5–25 mg), irradiation time (5–30 min), and H_2_O_2_ amount (0.2–2 mL) were selected for modeling and optimization of the photo-Fenton catalytic reaction. The experimental data obtained were fitted to different models and then analysis of variance (ANOVA) was applied to identify the best-fitting one.

### 2.8. Kinetic study

Pseudo-first-order (Eq. 2) and pseudo-second-order (Eq. 3) kinetic models were employed to analyze the photo-Fenton degradation of AB113 using MoS_2_/MgFe_2_O_4_:


(2) $$\text{ln}(\frac{C_{0}}{C})=k_{1}t+b$$

(3) $$\frac{1}{C}-\frac{1}{C_{0}}=k_{2}t$$

## 3. Results and discussion

### 3.1. Nanocomposite characterization

**Figure 1:**
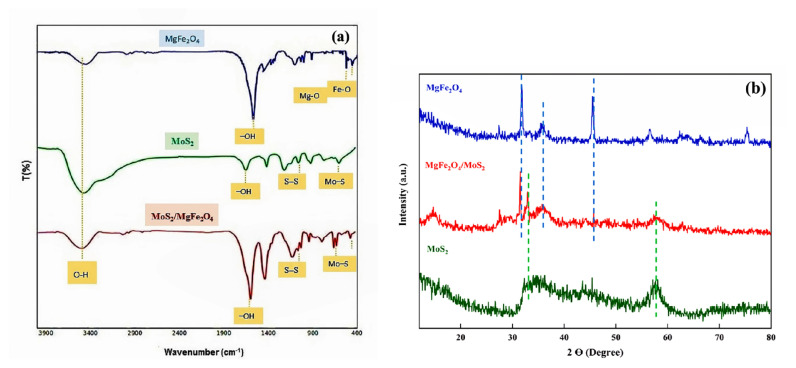
**(a)** FT-IR spectra and **(b)** XRD pattern of MgFe_2_O_4_ nanoparticles, the MoS_2_ nanostructure, and MgFe_2_O_4_/MoS_2_.

**Figure 2:**
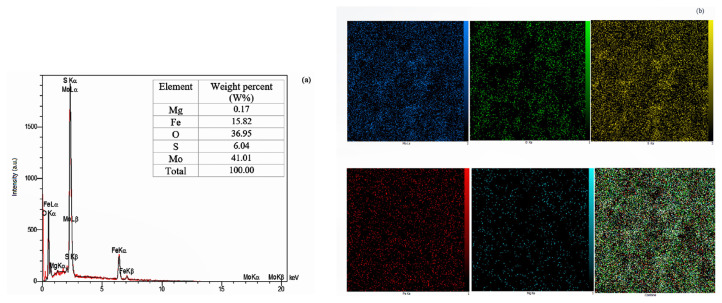
**(a)** EDS analysis and **(b)** map analysis of MgFe_2_O_4_/MoS_2_.

The FT-IR spectral characterization of MgFe_2_O_4_, MoS_2_, and MgFe_2_O_4_/MoS_2_ is illustrated in Figure 1. The peaks appearing at 460 cm^−1^ and 536 cm^−1^ are due to the stretching vibrations of Fe–O and Mg–O, respectively, demonstrating the formation of a spinel ferrite structure [24]. The peaks at 471 cm^−1^ and 617 cm^−1^ correspond to the Mo–S bonds’ stretching vibrations, whereas the peak at 1115 cm^−1^ is attributed to the S–S bond vibrations between neighboring layers of MoS_2_ [25]. Furthermore, the bands at 1575 cm^−1^ and 3458 cm^−1^ are related to the bending and stretching vibrations of surface O–H groups and the physical adsorption of water molecules. These findings offer compelling evidence of the successful fabrication of MgFe_2_O_4_/MoS_2_. Figure 2 displays the XRD patterns of MgFe_2_O_4_/MoS_2_, MgFe_2_O_4_ nanoparticles, and MoS_2_ nanostructure. The peaks located at 2θ = 31.8°, 35.75°, 45.5°, 56.55°, 62.38°, and 75.35° are attributed to (220), (311), (400), (422), (511), and (440) planes, respectively, thereby confirming the MgFe_2_O_4_ inverted spinel structure [22]. In addition, the XRD pattern of MoS_2_ shows two peaks at 2θ = 34.5° and 57°, related to the (100) and (110) planes, respectively [26]. The appearance of these peaks in the XRD pattern of MgFe_2_O_4_/MoS_2_ indicates that MoS_2_ and MgFe_2_O_4_ are present in the nanocomposite fabricated.

Figures 2a and 2b show the EDX and map analysis of the synthesized MgFe_2_O_4_/MoS_2_, respectively. EDX analysis confirms the existence of Mg, Fe, O, and Mo with no other additional contaminants present. According to the map analysis, the items are distributed uniformly.

**Figure 3:**
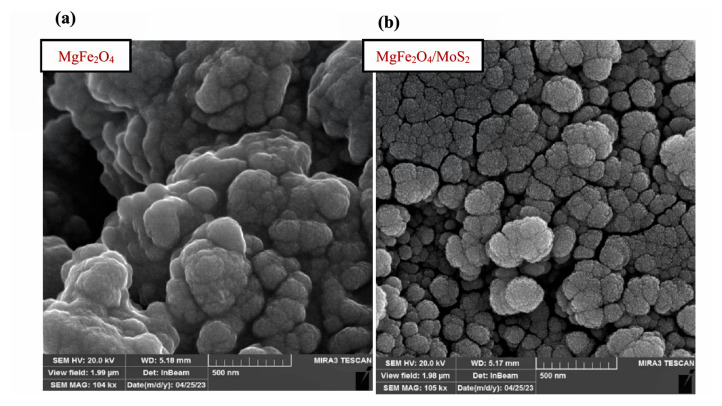
SEM images of **(a)** MgFe_2_O_4_ and **(b)** MgFe_2_O_4_/MoS_2_.

**Figure 4:**
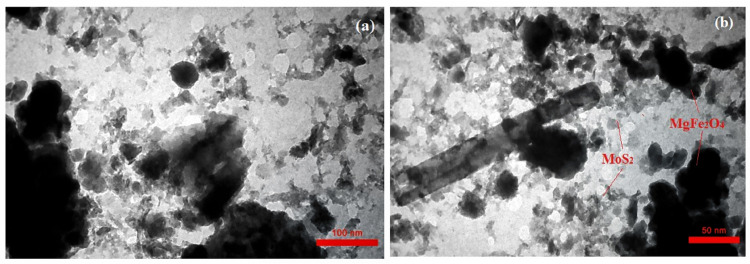
TEM images of MgFe_2_O_4_/MoS_2_ at different magnifications.

Figure 3a presents the SEM image of MgFe_2_O_4_, which shows that the particles are mostly spherical [27]. The SEM image of MgFe_2_O_4_/MoS_2_ is displayed in Figure 3b. Examination of the SEM image of MgFe_2_O_4_/MoS_2_ shows that MoS_2_ nanosheets surround ferrite particles. The ferrite particles are between 12 and 41 nm in size. Figure 4 shows the TEM images of MgFe_2_O_4_/MoS_2_ at different magnifications. As can be seen, MgFe_2_O_4_ nanoparticles are successfully anchored and distributed on the surface of the MoS_2_ nanosheets, confirming the formation of the composite structure. In the high-magnification image, some elongated rod-like features can be observed, which are associated with folded or restacked MoS_2_ nanosheets because of the layered and flexible nature of MoS_2_ during the composite formation process.

**Figure 5:**
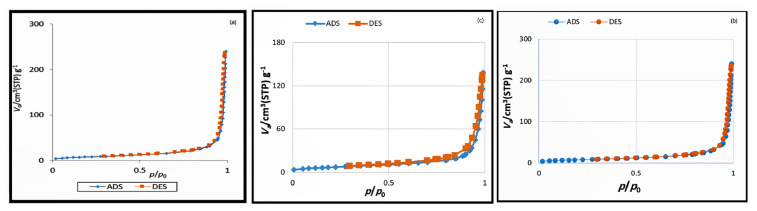
Adsorption–desorption isotherm of **(a)** MgFe_2_O_4_/MoS_2_, **(b)** MgFe_2_O_4_ nanoparticles, and **(c)** MoS_2_ nanostructure.

The adsorption–desorption isotherm of the nanocomposite is presented in Figure 5a. The type VI adsorption isotherm curve represents a mesoporous material with particle diameters of 2 to 50 nm. The BET physical parameters of the nanomaterials produced are given in Table 1 and the adsorption and desorption isotherms for MgFe_2_O_4_ and MoS_2_ nanoparticles are shown in Figures 5b and 5c, respectively. The presence of ferrite in the nanocomposite inhibits the stacking of the MoS_2_ layers, which results in enhanced surface area, pore diameter, and total pore volume, enabling better mass transfer.

**Figure 6:**
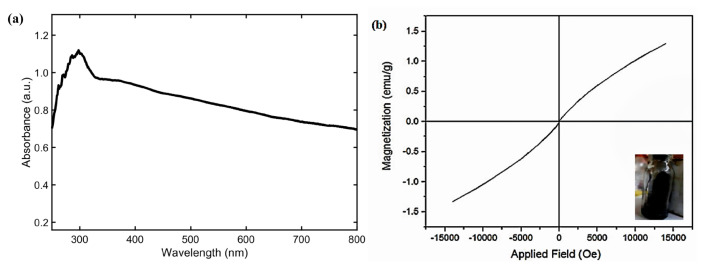
**(a)** The UV–Vis diffuse reflectance spectrum and (b) VSM curve of MgFe_2_O_4_/MoS_2_.

The UV–Vis diffuse reflectance spectrum of the nanocomposite (Figure 6a) exhibits a broad absorption band extending into the visible-light region, which may be due to the presence of MoS_2_ in the nanocomposite. This ability to absorb visible light supports the improved photo-Fenton performance of the nanocomposite under LED irradiation. Figure 6b shows the magnetic properties of MgFe_2_O_4_/MoS_2_. The maximum applied field (Hm) was 15,000 Oe. According to the result, the saturation magnetization value of MgFe_2_O_4_/MoS_2_ is 1.25 emu g^−1^. Although the presence of MoS_2_ decreases the magnetic properties of magnesium ferrite, the nanocomposite synthesized still has ferromagnetic properties [28].

### 3.2. Statistical analysis and model development

According to Table 2, the ANOVA study, the best-fitting model for describing AB113 removal is a quadratic model with the following equation (Eq. 4):


(4) $$\text{AB}113\;\text{removal }(\%)=+89.45+3.39\;\text{A}+1.99\;\text{B}-0.078\;\text{C}+1.03\;\text{D}+3.36\;\text{AB}+1.14\;\text{AC}-1.65\;\text{AD}-4.87\;{\text{A}}^{2}$$

**Figure 7:**
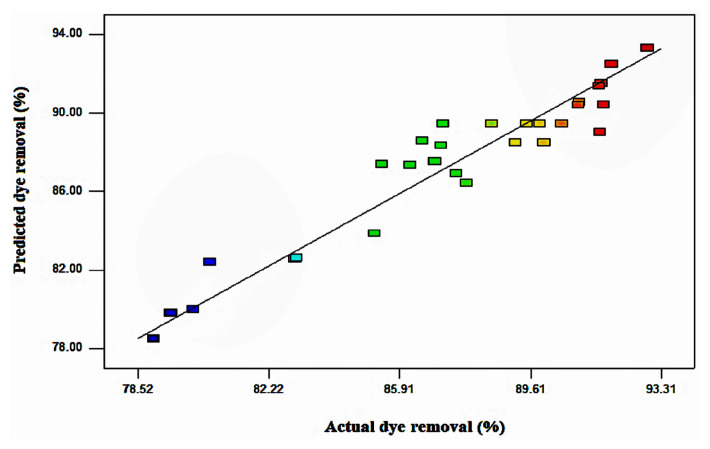
Correlation between the actual and predicted responses for the degradation of Acid Blue 113.

In this model, A represents the pH, B refers to H_2_O_2_ concentration, C is reaction time, and D stands for the amount of nanocatalyst. The coefficient of determination (R^2^) for this model is 0.9166, which is significant at the 99% confidence level (p > 0.0001). The result of variance analysis demonstrates that the chosen parameters have a significant effect on dye degradation. The agreement of the experimental and predicted dye degradation data with the model is illustrated in Figure 7.

### 3.3. Impact of operational parameters

**Figure 8:**
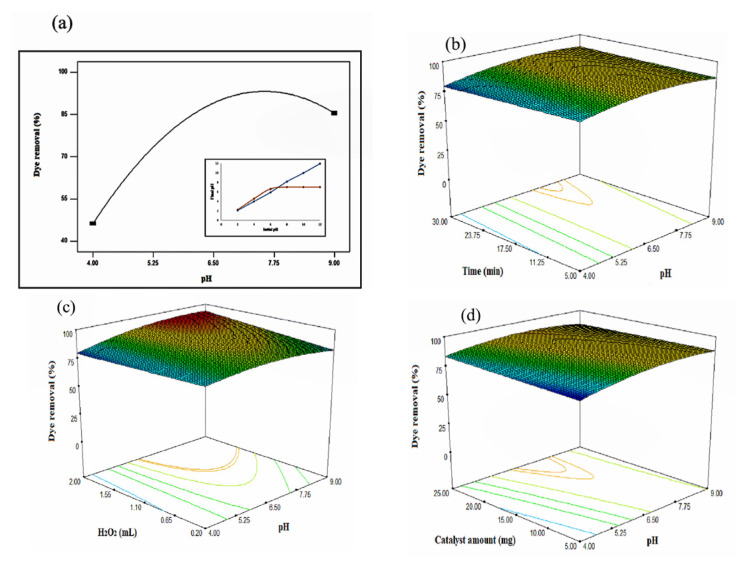
**(a)** Diagram of determination of the isoelectric point and interactive effects of **(b)** time and pH, **(c)** catalyst amount and pH, **(d)** H_2_O_2_ and pH.

The isoelectric point (pH_pzc_) of the nanocomposite was nearly 7, as seen in Figure 8a. At pH values below this level, the surface of the photocatalyst becomes positively charged, resulting in increases in the electrostatic attraction between AB113 anionic dye molecules and the catalyst surface, and hence enhancing dye removal efficiency. However, under highly acidic conditions, the hydroxide ion concentration is low and hydroxyl radical formation is limited; this causes the removal efficiency to decline. For pH values above the pH_pzc_, the catalyst surface becomes negatively charged, which leads to electrostatic repulsion between the catalyst surface and the anionic AB113 dye, and therefore the adsorption of the dye on the catalyst surface declines to some extent. However, under mildly alkaline conditions, the enhanced ^•^OH radicals compensate for the reduced dye adsorption and promote dye degradation through the photo-Fenton process. At higher pH values, excess hydroxide ions have the ability to absorb the ^•^OH radicals produced, resulting in a decrease in dye degradation [29]. Accordingly, the maximum degradation efficiency of AB113 can be achieved in the pH range of 7.5–8.8, where OH radical production and photo-Fenton activity are maximized.

As illustrated in Figure 8b, the effects of reaction time (5 to 30 min) and pH (4 to 9) on dye removal effectiveness were evaluated using the photo-Fenton method. The highest removal efficiency was attained at pH 8.79 with a reaction time of 27 min. The impact of time on dye removal is not significant, and a large amount of AB113 is removed in 5 to 10 min. Increasing the contact time at higher pH raised dye removal efficiency; this is explained by the increased production of hydroxyl radicals over time.

As shown in Figure 8c, the effect of catalyst dosage (5 to 20 mg) and pH (4–9) on the efficiency of AB113 dye removal in the reaction was investigated. The highest dye removal efficiency was attained at a catalyst dosage of 10 mg. Removal efficiency rose with increasing catalyst dosage up to the optimum value, which is ascribed to the greater availability of active sites for producing free radicals. By raising the amount of catalyst over the optimal amount, the solution becomes turbid and dye removal efficiency decreases due to limitations in mass transfer [30].

As shown in Figure 8d, the effects of H_2_O_2_ dosage (0.2 to 2 mL) and pH (4–9) on the efficiency of removing AB113 dye in the oxidation system were evaluated. The highest dye degradation percentage was attained at the optimal dosage of H_2_O_2_ (1.6 mL). The efficiency of dye removal improved with increasing H_2_O_2_ dosage up to the optimal value. Raising the amount of H_2_O_2_ beyond the optimum level prevents the formation of hydroxyl radicals, hence reducing dye removal efficiency [31].

In the present work, the optimum conditions for maximum AB113 degradation were predicted by RSM software. The optimal values obtained were a reaction time of 27 min, pH of 8.79, H_2_O_2_ dosage of 1.6 mL, and catalyst amount of 10 mg. Under the optimized conditions, the maximum dye degradation efficiency obtained was 94.92%.

### 3.4. Kinetic modeling of AB113 degradation

**Figure 9:**
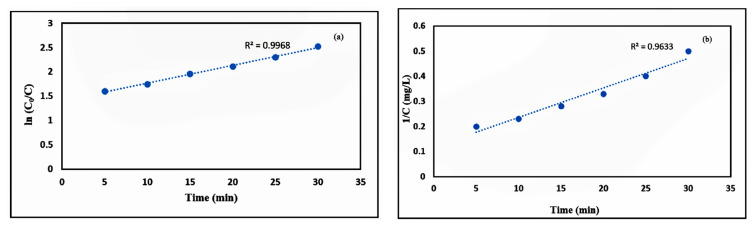
**(a)** Pseudo-first-order kinetic model and **(b)** pseudo-second-order kinetic model of AB113 degradation using MgFe_2_O_4_/MoS_2_.

**Figure 10:**
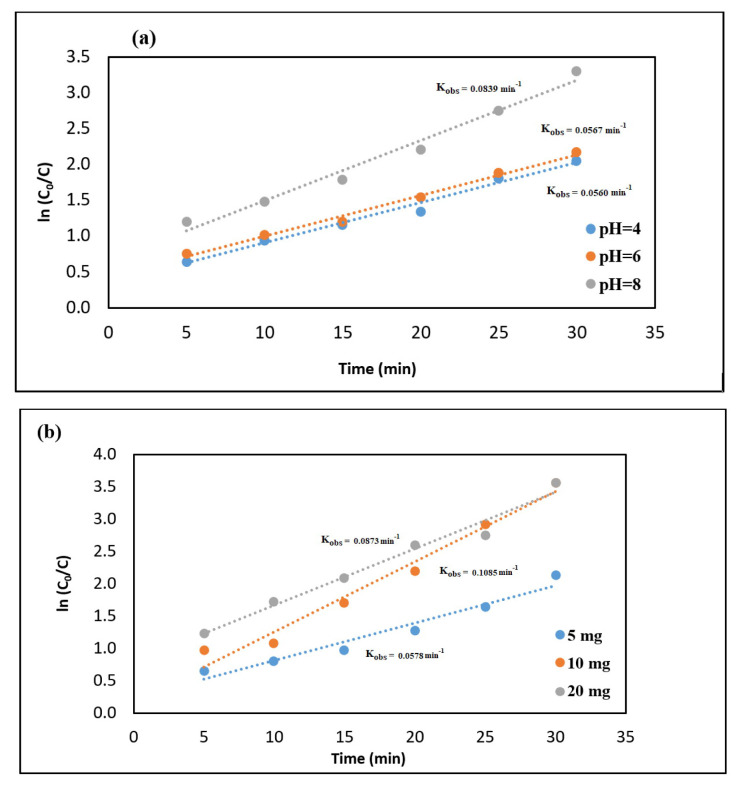
**(a)** Ln (C_0_/C) vs. time for various pH and **(b)** catalyst amounts.

The kinetics of AB113 degradation in the photo-Fenton oxidation process was investigated by applying pseudo-first-order and pseudo-second-order kinetic models, as presented in Figures 9a and 9b. From the results obtained, it can be concluded that the experimental data could fit well with the pseudo-first-order kinetic model having a high coefficient of determination (R^2^ = 0.997), which is consistent with previously reported studies on photo-Fenton processes employing different catalysts [18]. Accordingly, the rate constants (K_obs_) were determined from the slopes of the ln (C_0_/C) plots for various pH values and catalyst dosages. As can be seen in Figure 10a, the K_obs_ increases with increasing pH from 4 to 8. As mentioned previously, under mildly alkaline conditions, hydroxyl radical generation is enhanced, which consequently promotes the photo-Fenton activity. As illustrated in Figure 10b and also discussed previously, the maximum dye removal efficiency is obtained at a catalyst dosage of 10 mg, corresponding to the highest K_obs_ value.

### 3.5. Possible mechanism of AB113 degradation

In order to determine the ROS responsible for AB113 degradation by MgFe_2_O_4_/MoS_2_, radical scavenging experiments were performed using several scavengers, including oxalic acid (OA; h^+^ scavenger), benzoquinone (BQ; ^•^O_2_^−^ scavenger), sodium azide (NaN_3_;^1^O_2_ scavenger), and isopropyl alcohol (i-PrOH; ^•^OH scavenger) [32].

**Figure 11:**
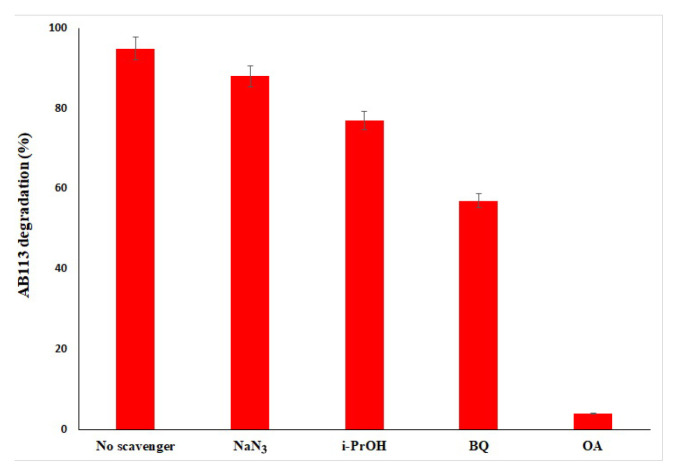
Effect of various scavengers on AB113 degradation.

The results obtained are presented in Figure 11. Under visible light irradiation, MoS_2_ enhances light absorption and increases the generation of electron-hole pairs, whereas MgFe_2_O_4_ provides active iron sites for Fenton reactions. The photogenerated holes play a crucial role in the photo-Fenton degradation of AB113, probably by promoting direct oxidation pathways and hydroxyl radical generation, whereas ^•^OH and superoxide radicals (^•^O_2_^−^) act synergistically in the overall degradation mechanism.

### 3.6. Reusability of MgFe_2_O_4_/MoS_2_

**Figure 12:**
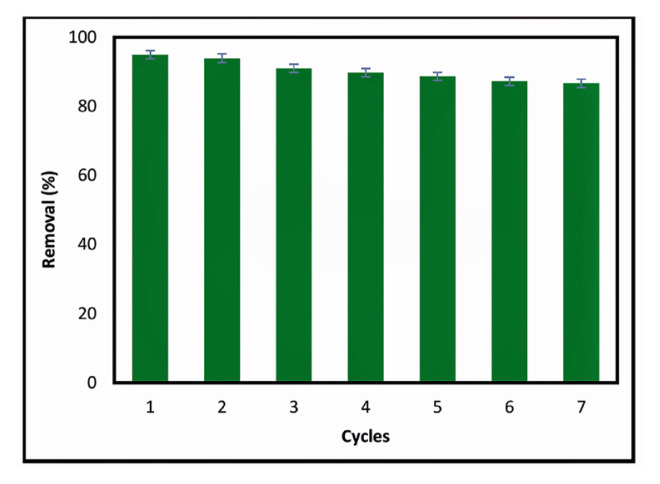
Reuse of MgFe_2_O_4_/MoS_2_ in removing Acid Blue 113.

To assess the stability of MgFe_2_O_4_/MoS_2_, degradation experiments were carried out under optimal conditions (27 min, pH 8.79, 1.6 mL of H_2_O_2_, and 10 mg of catalyst) for seven cycles. At each stage, the nanocomposite was extracted from the solution using a strong magnet and reused in the next run. According to Figure 12, the results demonstrate that the nanocomposite has good stability and reusability. After seven cycles, the removal efficiency for AB113 reached 86.66%. This decline in efficiency can be attributed to unavoidable nanocatalyst loss throughout the reuse cycles.

### 3.7. Evaluation of several oxidation systems’ dye degradation efficiency

The optimum dye degradation efficiency of the heterogeneous photo-Fenton approach (MgFe_2_O_4_/MoS_2_/H_2_O_2_/Vis) used in the present study is compared with that of a number of other advanced oxidation systems in Table 3. As can be seen, the proposed system demonstrates a superior dye degradation efficiency using a relatively low catalyst dosage.

As shown in Table 3, pure MgFe_2_O_4_ exhibited negligible activity, while MoS_2_ showed limited degradation efficiency under the optimized conditions found in the present study. The low activity of the individual components can be attributed to rapid charge carrier recombination and limited active sites. The synergistic interaction between MgFe_2_O_4_ and MoS_2_ in the composite can significantly improve the charge separation and photocatalytic activity of the nanocomposite synthesized.

## 4. Conclusions

In the present study, MgFe_2_O_4_ nanoparticles, MoS_2_ nanosheets, and the nanocompositeMgFe_2_O_4_/MoS_2_ were effectively synthesized via a facile hydrothermal approach and characterized. The results of FTIR and XRD analyses confirmed the coexistence MgFe_2_O_4_ and MoS_2_ within the nanocomposite’s structure. EDX analysis further verified the existence of the elements Mg, Fe, Mo, S, and O without impurities. SEM and TEM images indicated uniform dispersion of MgFe_2_O_4_ particles on the layered MoS_2_ sheets, while VSM results demonstrated the desirable ferromagnetic properties of the nanocomposite synthesized. The photo-Fenton-like process under optimal conditions (pH 8.79, 10 mg of catalyst, 1.6 mL of H_2_O_2_, and 27 min irradiation time) resulted in a maximum removal efficiency of 94.92%. The removal efficiency was strongly influenced by pH, catalyst dosage, and H_2_O_2_ concentration. This nanocatalytic system maintained an efficiency of 86.66% after seven reuses, indicating its high stability and recyclability. Overall, MgFe_2_O_4_/MoS_2_ shows significant potential as an effective and sustainable photo-Fenton catalyst for the degradation of organic dyes and other pollutants from industrial wastewaters.

## Figures and Tables

**Table 1:** BET physical parameters of MgFe_2_O_4_, MoS_2_, and MgFe_2_O_4_/MoS_2_.

Nanomaterial	BET surface area (m^2^ g^−1^)	Total pore volume (cm^3^ g^−1^)	Average pore diameter (nm)
MgFe_2_O_4_/MoS_2_	28.43	0.36	50.35
MoS_2_	7	0.02	12
MgFe_2_O_4_	11.8	0.14	48.96

**Table 2:** ANOVA of the model developed.

Source	Sum of squares	Degree of freedom	Mean square	*F*-value	p-value
Model	425.78	8	53.22	27.46	<0.0001
A, pH	137.50	1	137.50	70.94	<0.0001
B, H_2_O_2_ (mL)	47.44	1	47.44	24.48	<0.0001
C, time (min)	0.072	1	0.072	0.037	0.8490
D, catalyst dose (g)	12.75	1	12.75	6.58	0.0185
AB	45.09	1	45.09	23.27	0.0001
AC	5.15	1	5.15	2.66	0.1186
AD	10.86	1	10.86	5.60	0.0281
A^2^	166.92	1	166.92	86.12	<0.0001
Residual	38.76	20	1.94		
Lack of fit	31.88	16	1.99	1.16	0.4930
Pure error	6.88	4	1.72		
Corrected total	464.54	28			

**Table 3:** Comparison of dye degradation efficiency in different advanced oxidation processes.

Oxidation system	Reaction conditions	Efficiency (%)	Source
MgFe_2_O_4_.MoS_2_/H_2_O_2_/vis	AB113: 25 mg L^−1^, time: 26 min, catalyst: 10 mg, pH 8.79, H_2_O_2_ amount: 1.6 mL	94.92	Current research
MgFe_2_O_4_/vis	AB113: 25 mg L^−1^, time: 26 min, catalyst: 10 mg, pH 8.79	0	Current research
MoS_2_/vis	AB113: 25 mg L^−1^, time: 26 min, catalyst: 10 mg, pH 8.79	35	Current research
Fe_3_O_4_.MnO_2_.MoS_2_/O_3_/Vis	AB113: 25 mg L^−1^, time: 20 min, catalyst: 20 mg, pH 3, ozone flow 0.2: g L^−1^ h^−1^	99	[33]
ZnFe_2_O_4_/UV/H_2_O_2_	Methylene blue: 50 mg L^−1^, time: 120 min, catalyst: 10mg, pH 3, H_2_O_2_: 2.5 mM	88	[34]
ZnS-TiO_2_/UV	AB113: 25 mg L^−1^, time: 27 min, catalyst: 37 mg, pH 6.18	99.20	[35]
TiO_2_/β-FeOOH/Vis	Methyl orange: 80 mg L^−1^, time: 120 min, catalyst: 20 mg, pH 4.5	86	[36]
